# ANT2-Mediated ATP Import into Mitochondria Protects against Hypoxia Lethal Injury

**DOI:** 10.3390/cells9122542

**Published:** 2020-11-25

**Authors:** Yves Gouriou, Muhammad Rizwan Alam, Zeina Harhous, Claire Crola Da Silva, Delphine Baetz Baetz, Sally Badawi, Etienne Lefai, Jennifer Rieusset, Annie Durand, Rania Harisseh, Abdallah Gharib, Michel Ovize, Gabriel Bidaux

**Affiliations:** 1 Univ-Lyon, CarMeN Laboratory, INSERM 1060, INRA 1397, Université Claude Bernard Lyon1, INSA Lyon, Oullins, France, IHU OPERA, Groupement Hospitalier EST, Bâtiment B13, 59 boulevard Pinel, F-69500 Bron, France; rizwan805@gmail.com (M.R.A.); zeina.harhous@gmail.com (Z.H.); claire.crola-da-silva@univ-lyon1.fr (C.C.D.S.); delphine.baetz@univ-lyon1.fr (D.B.B.); sally_b86@hotmail.com (S.B.); Etienne.Lefai@inra.fr (E.L.); jennifer.rieusset@univ-lyon1.fr (J.R.); annie.durand@univ-lyon1.fr (A.D.); rania.harisseh@gmail.com (R.H.); abdallah.gharib@univ-lyon1.fr (A.G.); michel.ovize@gmail.com (M.O.); 2 Department of Biochemistry, Quaid-i-Azam University, Islamabad 45320, Pakistan; 3 Gilbert and Rose-Marie Chagoury, School of Medicine, Lebanese American University, Byblos 4M8F+8X, Lebanon

**Keywords:** mitofusin 2, mitochondria-associated membranes, hypoxia, metabolism, bioenergetics, ATP, ANT2, ATP synthase, IF1, mitochondrial membrane potential

## Abstract

Following a prolonged exposure to hypoxia–reoxygenation, a partial disruption of the ER-mitochondria tethering by mitofusin 2 (MFN2) knock-down decreases the Ca^2+^ transfer between the two organelles limits mitochondrial Ca^2+^ overload and prevents the Ca^2+^-dependent opening of the mitochondrial permeability transition pore, i.e., limits cardiomyocyte cell death. The impact of the metabolic changes resulting from the alteration of this Ca^2+^crosstalk on the tolerance to hypoxia–reoxygenation injury remains partial and fragmented between different field of expertise. >In this study, we report that MFN2 loss of function results in a metabolic switch driven by major modifications in energy production by mitochondria. During hypoxia, mitochondria maintain their ATP concentration and, concomitantly, the inner membrane potential by importing cytosolic ATP into mitochondria through an overexpressed ANT2 protein and by decreasing the expression and activity of the ATP hydrolase via IF1. This adaptation further blunts the detrimental hyperpolarisation of the inner mitochondrial membrane (IMM) upon re-oxygenation. These metabolic changes play an important role to attenuate cell death during a prolonged hypoxia–reoxygenation challenge.

## 1. Introduction

Despite major progress over the past decades, morbidity following acute myocardial infarction remains too high and a better understanding of mechanisms of cell death after ischemia–reperfusion is needed to improve therapy of acute coronary syndromes. Mitochondrial calcium overload plays a crucial role in myocardial ischemia–reperfusion injury in particular via the activation of the Ca^2+^-dependent cyclophilin D-mediated opening of the mitochondrial permeability transition pore [1]. In-vitro studies, alteration of ER-to-mitochondrial calcium homeostasis in conjunction with oxidative stress have been reported to open the mitochondrial permeability transition pore (mPTP). In turn, this causes both collapse of the mitochondrial membrane potential and swelling of mitochondria. Consequently, pyridine nucleotides and apoptosome components are released while the bioenergetics failure leads the cells to necrotic cell death [2,3,4]. Several groups have also shown that mitochondrial calcium, despite its undirect role in necrosis, could also coordinate various levels of the apoptotic process [5,6]. Mitochondrial calcium largely originates from the ER, trafficking through membrane contact points referred to as mitochondrial-associated membranes (MAMs) [7]. Mitochondrial matrix Ca^2+^ is however not only a trigger of the opening of the permeability transition pore (PTP), but is also a regulator of FAD-glycerol phosphate dehydrogenase, pyruvate dehydrogenase, NAD-isocitrate dehydrogenase and oxoglutarate dehydrogenase of the Krebs cycle (for review see, [8]).

We previously reported that a concomitant disruption of the Ca^2+^ transfer from ER to mitochondria through the IP3R-VDAC complex at MAMs decreases cardiomyocyte death after a prolonged hypoxia–reoxygenation [9]. However, we did not specifically address how this perturbation of ER-mitochondria crosstalk could also involve an energy crisis and its consequences in cell tolerance to the hypoxia–reoxygenation induced injury.

Cancer cells that survive in the hypoxic environment of the tumor core display a low MFN2 expression [10,11,12,13,14,15]. In in vivo conditions of hypoxia–reoxygenation, Hall et al. reported that hearts of MFN2^-/-^ mice are more tolerant to acute myocardial infarction. Beyond its role in mitochondria fusion, mitofusin 2 (MFN2) contributes to the tethering of these two organelles, stabilizes MAMs and facilitates Ca^2+^ shuttling between the two organelles [16,17]. Depletion in MFN1/MFN2 expression induces the transcription of genes involved in glycolytic metabolism via hypoxia-inducible factor 1α [18]. MFN2^-/-^mitochondria exhibit a metabolic reprogramming towards glycolysis instead of aerobic metabolism (OXPHOS) [19]. We then questioned whether a modification of the cell bioenergetics secondary to MFN2 loss of function might contribute to the survival of cardiomyocytes exposed to hypoxia–reoxygenation.

In the present study, we showed that a transient decrease in MFN2 expression enhanced the tolerance to hypoxia–reoxygenation via a metabolic reprogramming including a decreased mitochondria OXPHOS activity together with an enhanced anaerobic glycolysis. This adaptative metabolic switch involves both an unexpected import of cytosolic ATP into mitochondria via ANT2 and a decrease in F_1_F_0_ ATP hydrolase activity that is caused by an increased expression of its inhibitor, IF1.

## 2. Results

### 2.1. Increased Mitochondrial ATP Despite Reduced Ca^2+^ Accumulation after MFN2 Loss of Function

**Figure 1:**
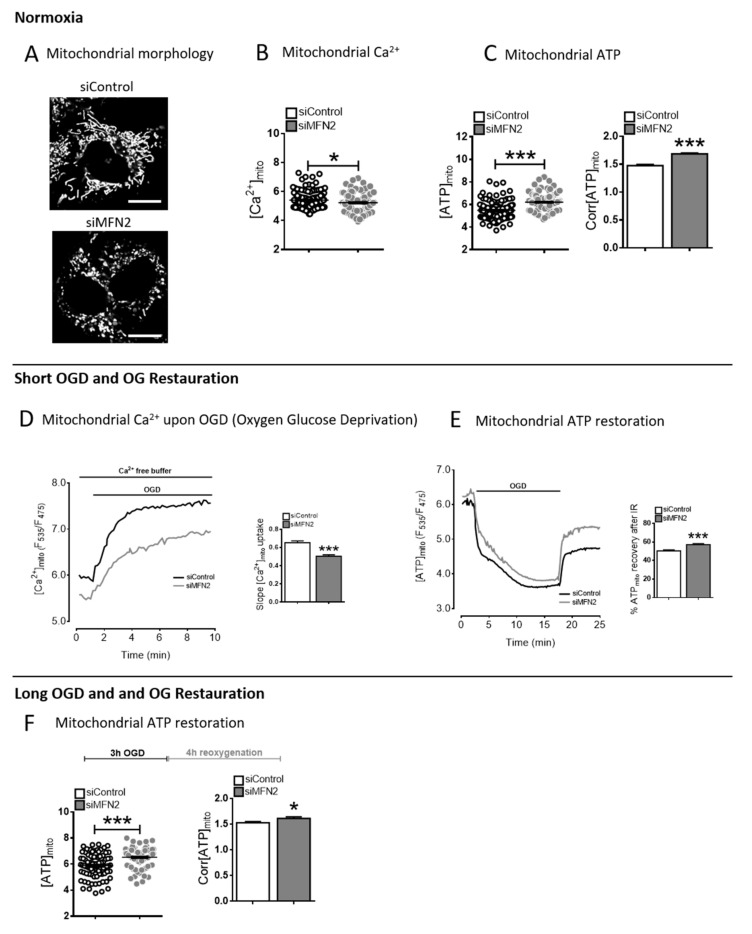
The Calcium/ATP paradox in mitofusin 2 (MFN2)-KD cells. (**A**) Confocal images of H9C2-sv40 cells expressing the mitochondrial Ateam sensor in siControl and MFN2-KD conditions (Scale bars, 10 μm). (**B**) Dot plot of steady-state mitochondrial Ca^2+^ level in siControl (black) and siMFN2 (grey) H9C2-sv40 cells (F_535_/F_475_). *n* = 125, 116 cells for siControl and siMFN2 H9C2-sv40 cells, respectively. Data shown represent the mean ± SEM of 4 independent experiments. * *p* < 0.05. (**C**) (left inset) Dot plot of steady-state mitochondrial ATP level in control (black) and MFN2-KD (grey) H9C2-sv40 cells (F_535_/F_475_). (right inset) Corrected steady-state mitochondrial ATP level (Corr[ATP]_mito_) calculated as ratio of Ateam values before/after 2-Deoxyglucose and oligomycin A for each cell (see Figure S2). *n* = 126, 139 cells respectively. Data shown represent the mean ± SEM of 4 independent experiments. (**D**) Mitochondrial calcium measurement during oxygen glucose deprivation (OGD) in siControl (black) and siMFN2 (grey) H9C2-sv40 cells. Maximal slope analysis of mitochondrial Ca^2+^ entry upon OGD in H9C2-sv40 cells. *n* = 125, 116 cells for siControl and siMFN2 H9C2-sv40 cells, respectively. Data shown represent the mean ± SEM of 4 independent experiments. *** *p* < 0.001. These experiments have been performed in absence of external calcium. (**E**) Measurement of the mitochondrial ATP level during a 15 min-oxygen glucose deprivation (OGD) and a 5 min-reoxygenation with glucose (reox + Glucose), in siControl (black) and siMFN2 (grey) H9C2-sv40 cells. Inset: Percentage of mitochondrial ATP recovery calculated as Ateam ratioat reperfusion divided by its steady-state value in siControl (white) and siMFN2 (grey) H9C2-sv40 cells. *n* = 85, 164 cells for siControl and siMFN2 H9C2-sv40 cells, respectively. Data shown represent the mean ± SEM of 4 independent experiments. (**F**) Measurements of steady-state mitochondrial ATP level after a 3 h-OGD and a 4 h-reoxygenation with glucose (reox + Glucose). (left inset) Dot-plot shows the steady-state mitochondrial ATP level ([ATP]_mito_) in each cell (F_535_/F_475_). (right inset) Corrected steady-state mitochondrial ATP level (Corr[ATP]_mito_). *n* = 75 cells for siControl and siMFN2 H9C2-sv40 cells. Data shown represent the mean ± SEM of 3 independent experiments, * *p* < 0.05; *** *p* < 0.001.

In normoxia conditions, we confirmed that the Ca^2+^content in mitochondria was lower in MFN2-KD H9C2-sv40 cells than in control cells (siControl) (Figure 1A,B; protein silencing is reported in Figure S1). It has been reported in several cell models that MFN2-KD may modify the contact surface between mitochondria and ER [20,21,22] and consequently perturbates Ca^2+^ transfer between ER and mitochondria. However, MFN2 KD is also known to modify mitochondria biogenesis and we questioned whether changes in mitochondrial volume and organization could also have an effect on the ER-to-mitochondria exchange surface. We labeled the ER and mitochondria of H9C2 cells with D1ER and MitoTracker DeepRed, respectively (Figure S2D). Pearson’s (data not sown) and Manders’s (data not shown) co-localization coefficients reported the concomitant detection of both fluorescence in pixels of images of either CTL or MFN2-KD H9C2 cells. In line with several studies [20,21,22,23,24], we detected a reduction in the co-localization of the two fluorescent signals in MFN2-KD cells which could be due either to a decrease in the proximity between ER and mitochondria membranes or a decrease in the surface of membrane-membrane contacts. However, pixel-based analysis (i.e., Manders’ and Pearson’s colocalization coefficients) only reports the proportion of one fluorescent signal that co-localizes with the second fluorescent signal. It does not preserve and quantify the spatial information of the signal. We estimated their density and surface area by mean of image correlation spectroscopy. In MFN2-KD cells, mitochondria surface area and MAMs surface area were unchanged while their density dropped by 1.8-fold (Figure S2E) and 1.5-fold (Figure S2F), respectively. Strikingly, the total surface area of the MAMs and mitochondria compartments decreased concomitantly in MFN2 KD cells (Figure S2E,F). This result may be of importance when one would interpret changes in protein expression level detected in a cell population lysate (i.e., Western-blot) but would have no artefactual effect for any ratiometric measurement single cell or single organelle.

We next measured mitochondrial ATP levels using the “genetically-encoded ATP indicator ATeam” which enables single live cell analysis. The mitochondrial ATP content was significantly higher in MFN2-KD cells averaging 6.209 ± 0.069 ∆F versus 5.485 ± 0.069 ∆F in Control cells. Normalized fluorescence ratio (values at origin over its value after the treatment with inhibitors of glycolysis and ATP synthase) gave a similar result showing a higher steady-state mitochondrial ATP value of 1.689 ± 0.014 ∆F in MFN2-KD cells than in Control cells of 1.477 ± 0.02171 ∆F (Figure 1C and Figure S2A,B).

Cells were then exposed to hypoxia OGD (oxygen glucose deprivation). We confirmed our previous results [9] showing that loss of MFN2 function prevented Ca^2+^ accumulation in mitochondria following a 10-min OGD (oxygen glucose deprivation) (Figure 1D). As expected, the 10-min OGD depleted the ATP content in mitochondria (Figure 1E) and cytosol (Figure S2C) of CTL andMFN2-KD cells. ATP content following oxygen-glucose reperfusion was significantly higher in mitochondria of MFN2-KD cells following both a short (10 min OGD) and a prolonged hypoxia (3 h OGD) (Figure 1E,F). We hypothesized that this could be explained either by an increase in OXPHOS-mediated ATP production or by a non-canonical ATP import from cytosol into mitochondria.

Overall, our results showed that, at baseline, mitochondria of MFN2-KD cells had an unexpected higher ATP concentration despite a lower Ca^2+^ content. Importantly, upon OGD MFN2-KD cells accumulated less Ca^2+^ than control cells and maintained a greater ATP level.

We then questioned the unexpected pattern upon OGD of a concomitant limited accumulation of mitochondrial calcium and an enhanced ATP production. To investigate this intriguing feature, we measured mitochondrial respiration and cell bioenergetics in MFN2-KD cells.

### 2.2. Decreased MFN2 Expression Triggers a Shift Toward a Cytosolic ATP Import into Mitochondria

**Figure 2:**
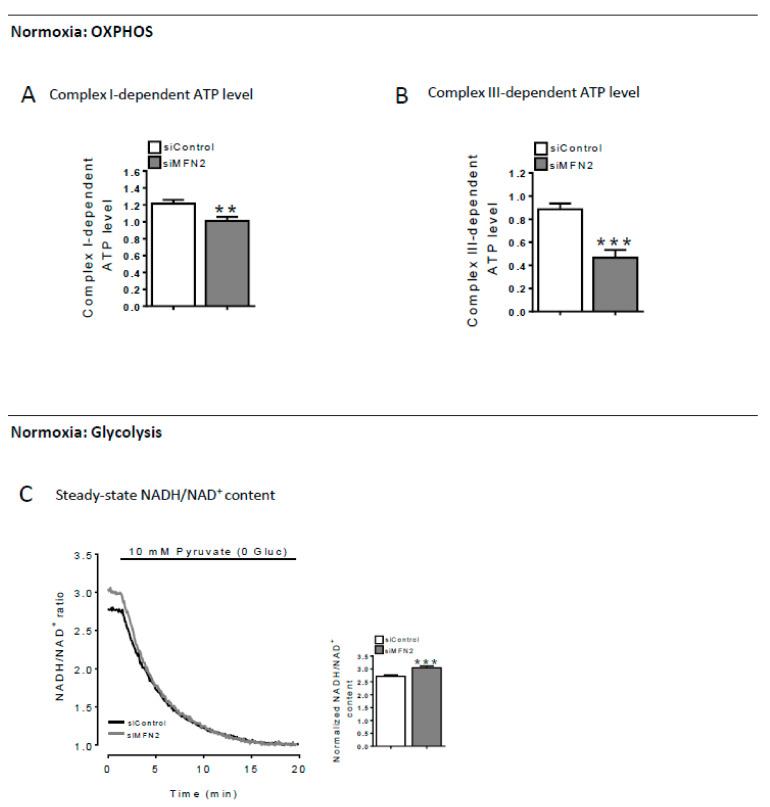
Decreased MFN2 expression induces a glycolytic phenotype. (**A**) Measurements of mitochondrial ATP level before and after a 1 µM rotenone treatment was performed in order to figure out the proportion of complex I-dependent ATP production. *n* = 40, 46 cells for siControl and siMFN2 H9C2-sv40 cells, respectively. Data shown represent the mean ± SEM of 3 independent experiments (See method details for the formula). (**B**) same as (**A**) with a 500 nM antimycin A treatment to calculate the proportion of complex III-dependent ATP level in the mitochondria. *n* = 20, 21 cells for siControl and siMFN2 H9C2-sv40 cells, respectively. Data shown represent the mean ± SEM of 3 independent experiments. (**C**) NADH/NAD+ ratio measurements obtained in control (black) and MFN2-KD (grey) H9C2-sv40 cells. A 10mM Pyruvate treatment, in absence of glucose, is achieved to determine the minimal NADH/NAD+ ratio value corresponding to the NADH insensitivity threshold of the probe (free NADH is consumed by the lactate dehydrogenase). (inset) Value represent mean ± SEM variation of NADH/NAD+ ratio between time 0 and post-pyruvate treatment (Normalized NADH/NAD+ ratio) obtained from 4 independent experiments. *n* = 81, 115 cells for siControl and siMFN2 H9C2-sv40 cells, respectively, ** *p* < 0.01, *** *p* < 0.001.

Activity of the OXPHOS complexes was monitored in live single cells at 37 °C under normoxia. Using a combination of inhibitors of complexes I and III and the A-team sensor, we detected a 1.3 and 1.9-fold decrease in Complex I- and Complex III-dependent ATP synthesis in MFN2-KD cells, that paralleled the decrease in Ca^2+^ concentration (Figure 2A,B). Obviously, the increased ATP concentration in mitochondria could not be due to the reduced OXPHOS activity.

We also assessed the glycolytic state of MFN2-KD cells by measuring the baseline ratio (reduced over oxidized) of nicotinamide adenine dinucleotide (NADH-NAD^+^) using a cytosolic fluorescent biosensor [25]. As reported in Figure 2C, we found a significantly higher NADH/ NAD^+^ ratio in MFN2-KD (3.053 ± 0.061∆F) when compared to control cells (2.711 ± 0.054 ∆F), consistent with an increased glycolysis [26,27,28,29].

**Figure 3:**
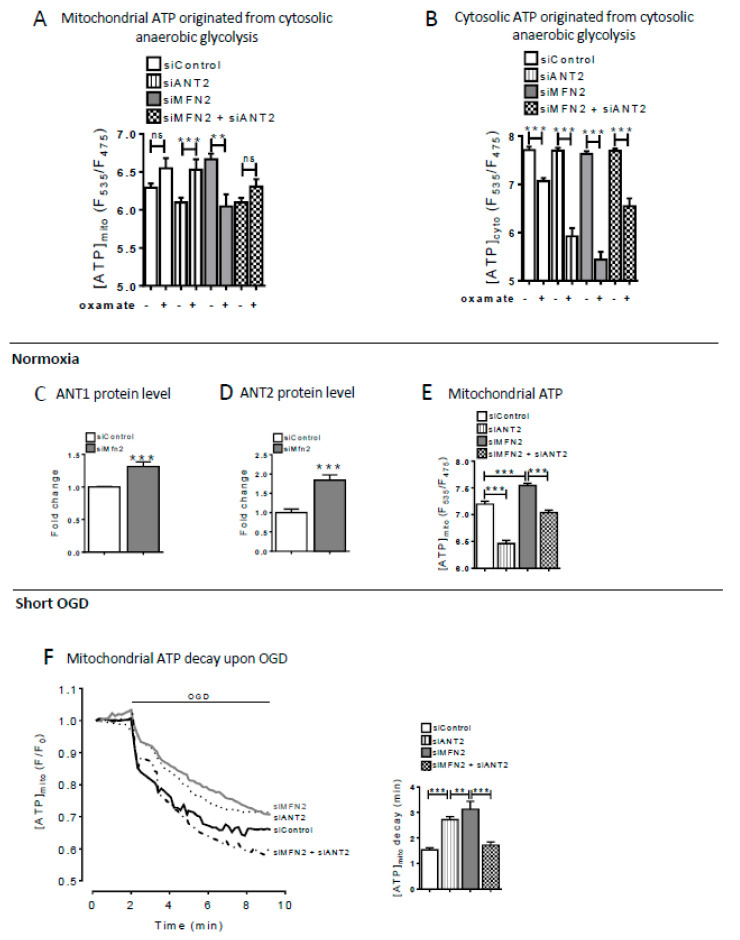
MFN2 loss of function induces an ANT2-dependent ATP import into mitochondria. (**A**) Estimation of the proportion of mitochondrial ATP originated from the cytosolic anaerobic glycolysis determined by mitochondrial ATP measurements in basal condition and after incubation with oxamate (20 mM) in control (white), siANT2 (stripped) MFN2-KD (grey) and siMFN2 + siANT2 (dashed) H9C2-sv40 cells. *n* = 125, 113, 122, 103, 106, 97, 104, 117 cells for siControl, siMFN2 and siMFN2 + siANT2 H9C2-sv40 cells, respectively. Data shown represent the mean ± SEM of 3 independent experiments. (**B**) Estimation of the proportion of cytosolic ATP originated from the cytosolic anaerobic glycolysis determined by cytosolic ATP measurements in basal condition and after incubation with oxamate (20 mM) in the cytosol of control (white), siANT2 (stripped), MFN2-KD (grey) and siMFN2 + siANT2 (dashed). *n* = 150, 195, 157, 121, 154, 131, 188, 134 cells for siControl, siMFN2 and siMFN2 + siANT2 H9C2-sv40 cells, respectively. Data shown represent the mean ± SEM of 3 independent experiments. ** *p* < 0.01; *** *p* < 0.001. (**C**) Western-blot showing ANT1 protein level in siControl (white) and siMFN2 (grey) H9C2-sv40 cells. ANT1 expression was normalized by the one of Tom20. *** *p* < 0.001 (see S4A for blot). (**D**) Western-blot of ANT2 protein normalized to Tom20 protein in siControl (white) and siMFN2 (grey) H9C2-sv40 cells. Data shown represent the mean ± SEM of 3 independent experiments (see S4B for blot). (**E**) Measurement of steady-state mitochondrial ATP in siControl, siANT2, siMFN2 and siMFN2 + siANT2 transfected cells. *n* = 226, 226, 188, and 217. Data shown represent the mean ± SEM of 3 independent experiments (**F**) Decay of the mitochondrial ATP content was measured by mean of mito-Ateam during oxygen glucose deprivation (OGD), in siControl (black), siANT2 (dotted), siMFN2 (grey) and siMFN2 + siANT2 (dashed) H9C2-sv40 cells. (inset) Mean lifetime of an exponential decay fit for mitochondrial ATP evoked by OGD in siControl, siANT2, siMFN2 and siMFN2 + siANT2. *n* = 153, 19, 231, and 89 cells. Data shown represent the mean ± SEM of 3–4 independent experiments. ** *p* < 0.01; *** *p* < 0.001.

We measured the effect of the LDH blocker oxamate on mitochondrial and cytosolic ATP levels under normoxia conditions (Figure 3A,B) and found that both cytosolic ATP and mitochondrial ATP concentration were more dependent on anaerobic glycolysis in MFN2-KD cells than in CTL cells. We checked whether this mechanism would be specific to H9C2-sv40 cells; we then repeated these experiments in a mouse cell line exhibiting typical hepatocyte features (AML12) and a mouse embryonic fibroblast cell line (MEF) (Figures S3A,B and S1C,D for shRNA validation). We obtained very comparable results suggesting that a transient suppression of MFN2 induces a shift of the molecular network toward a glycolytic metabolic phenotype in cell lines of different origins. We next investigated the mechanism that would allow ATP import from cytosol to mitochondria in MFN2KD cells.

### 2.3. Role of ANT2 for ATP Import into Mitochondria after MFN2 Loss of Function

Under normoxic conditions, MFN2-KD cells exhibited a near two-fold increase of ANT2 expression while ANT1expression increased slightly of 1.3 fold as compared to control (Figure 3C,D and Figure S4A,B). Regarding the decrease of the mitochondrial compartment in MFN2 KD cells (Figure S2E), these values could be underestimated. We have chosen to focus on ANT2 because it showed a greater induction and because studies reported that mitochondrial ATP import could be more dependent of ANT2 [30]. siRNA knocking down of ANT2 by 50% (Figure S4C) resulted in a significant decrease in mitochondrial ATP both in MFN2 KD and in CTL cells (Figure 3E) indicating a crucial role of ANT2 for glycolytic ATP import into mitochondria.

Pharmacological blockade of glycolysis by oxamate reduced cytosolic but not mitochondrial ATP content in control cells (Figure 3A,B). When oxamate was administered in siANT2 control cells, cytosolic ATP decreased but mitochondrial ATP increased. This indicates as expected that in control cells mitochondrial ATP is mainly synthesized in mitochondria and is exported into the cytosol. Conversely in MFN2 KD cells, both cytosolic and mitochondrial ATP content was reduced by oxamate. Additional loss of ANT2 function partially corrected the decrease in cytosolic ATP but did not significantly modify mitochondrial ATP content. This suggests that ANT2 imports cytosolic ATP into mitochondria in these cells and that unlike control cells, ANT2 plays a crucial role in MFN2-KD cells for importing cytosolic ATP formed by glycolysis into mitochondria.

We then questioned whether this metabolic shift would contribute to enhance the resistance of the cells to hypoxia–reoxygenation. We specifically explored the preservation of ATP using the OGD challenge. As expected, a transient OGD caused a rapid decrease of mitochondrial ATP in CTL cells (Figure 3F). MFN2 KD cells (ATP mean: 3.123 ± 0.311 min) exhibited a slower decrease in mitochondrial ATP content than CTL cells (ATP mean: 1.541± 0.085 min) (Figure 3F). Suppression of ANT2 alone significantly attenuated the decrease of mitochondrial ATP indicating that ANT2 either exports ATP from mitochondria to cytosol during OGD or that ANT2 favors ATP hydrolysis during OGD. As opposed to CTL cells, suppression of ANT2 accelerated, but not slowed down, the rate and amplitude of the decrease of mitochondrial ATP content in MFN2KD cells (Figure 3F) suggesting that ANT2 plays an important role for preserving mitochondrial ATP content during OGD. Altogether our data suggest that, during OGD, either ANT2 acts in a reverse mode (i.e., imports ATP into mitochondria) in MFN2KD cells or attenuate ATP hydrolysis during OGD in MFN2KD cells.

During hypoxia–reoxygenation, mitochondrial ATP hydrolysis occurs via the F_1_F_0_ ATPase or other catabolic enzymatic processes (i.e., kinases). We then assessed the activity of the F_1_F_0_ ATPase as well as the other ATP catabolic processes during OGD using the mitochondrial F_1_F_0_ ATPase inhibitor: BMS-199264 [31].

**Figure 4:**
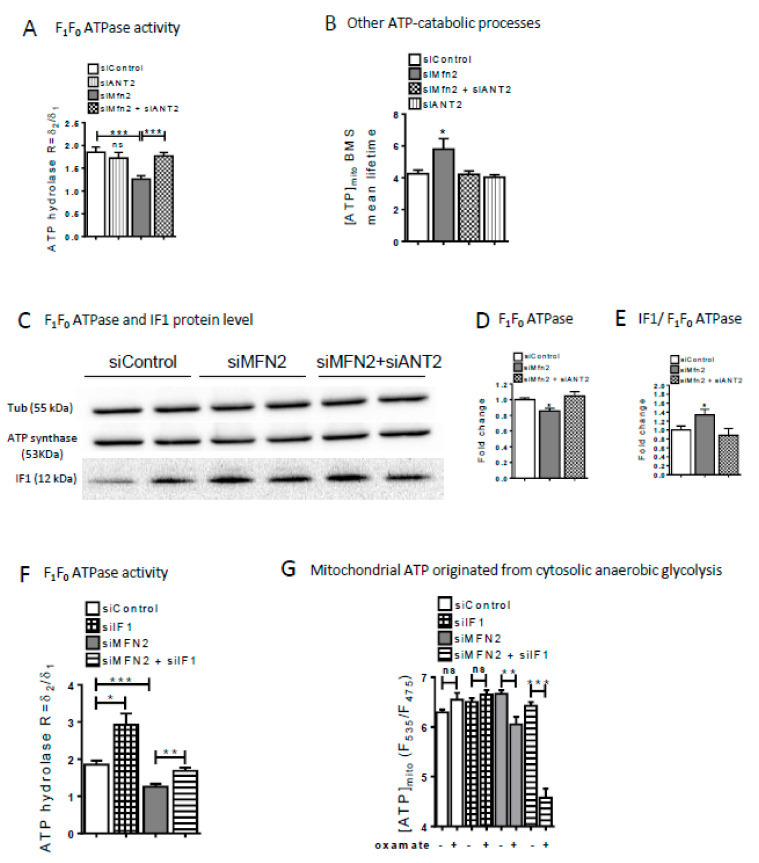
MFN2 loss upregulates ATPase inhibitory factor 1. (**A**) Estimation of F_1_F_0_ ATP hydrolase activity calculated as the difference in mitochondrial ATP decay during OGD with or without the F_1_F_0_ ATP hydrolase inhibitor BMS-199264 (10 µM) in siControl, siANT2, siMFN2, and siMFN2+siANT2 transfected cells. *n* = 48, 20, 62, and 48 cells. Mean lifetime of an exponential decay fit is used to calculate the mitochondrial ATP decay during OGD. δ1 = Mean lifetime in control condition and δ2 = Mean lifetime in presence of BMS. Data shown represent the mean ± SEM of 4 independent experiments. (**B**) Histogram showing the contribution of other ATP-catabolic processes during OGD in presence of F_1_F_0_ATP hydrolase inhibitor BMS-199264in H9C2-sv40 cells. Activity of these other ATP catabolic processes was quantified as the averaged mitochondrial ATP decay figured out by the mean lifetime of an exponential decay fitting the Ateam sensor fluorescence ratio over time. *n* = 65 (control), 73 (siMFN2), 48 (siMFN2 + siANT2) and 20 (siANT2) cells. Data shown represent the mean ± SEM of 3–4 independent experiments * *p* < 0.05. (**C**) Western-blot (WB) of ATP5a and IF1 protein normalized to Tubulin protein in siControl (white), siMFN2 (grey) and siMFN2+siANT2 (grey dashed line) in H9C2-sv40 cells. (**D**) Fold change of ATP5a WB density normalized to Tubulin protein represents the variation in ATP5a protein expression. (**E**) Fold change of IF1 WB density normalized to ATP5a protein represents the variation in negative regulatory IF-1 protein expression as compared to F_1_F_0_ATP synthase/hydrolase activity. Data shown represent the mean ± SEM of 4 independent experiments. * *p* < 0.05. (**F**) ATP hydrolase activity (calculated as explain in the Figure 4A) in siControl, siIF1, siMFN2, siMFN2 + siIF1, and siIF1 transfected cells. *n* = 48, 30, 62, and 48 cells. δ1 = Mean lifetime in control condition and δ2 = Mean lifetime in presence of BMS. Data shown represent the mean ± SEM of 4 independent experiments. (**G**) Estimation of the proportion of mitochondrial ATP originated from the cytosolic anaerobic glycolysis. Mitochondrial ATP measurements were achieved with mito-Ateam sensor expressed in basal condition and after incubation with oxamate (20 mM) in control, IF1, MFN2-KD and siMFN2 + siIF1 transfected H9C2-sv40 cells. *n* = 125, 113, 100, 106, 106, 97, 96, 84 cells, respectively. Data shown represent the mean ± SEM of 3 independent experiments * *p* < 0.05, ** *p* < 0.01; *** *p* < 0.001.

Loss of ANT2 had no effect on the F1Fo ATP hydrolase activity in CTL cells (Figure 4A). As shown previously in Figure 3F, MFN2 KD cells exhibited a decreased ATP hydrolase activity that was prevented by the concomitant suppression of ANT2, suggesting that ANT2 activity influences mitochondrial ATP hydrolysis during OGD in MFN2 KD cells. Non-F_1_F_0_ ATPase degradation processes were estimated by using BMS-199264. This inhibitor increased the mean lifetime of mitochondrial ATP content in MFN2 KD when compared to CTL cells indicating that a relative greater activation of these ATP catabolic processes in MFN2KD versus CTL cells (Figure 4B). Overall, ANT2 appears to play a dual role for preserving mitochondrial ATP content during OGD, i.e., facilitating its import and limiting its hydrolysis.

### 2.4. Decreased MFN2 Expression Up-Regulates the F1FoATPase Inhibitory Factor 1 and Maintain Mitochondrial Membrane Potential

The physiological ATPase inhibitory factor 1 (IF1) prevents the ATPase to switch to its ATP hydrolase form when the mitochondrial electrochemical gradient collapses as a consequence of hypoxia [32]. In cancer cells, the up-regulation of IF1 prevents a useless waste of ATP and is related to a Warburg-like metabolic shift [33].

Under normoxic conditions, MFN2-KD cells displayed a 25% decreased expression of the ATPase (ATP5a)—which could be related to the 25% decrease in the total mitochondrial compartment (Figure S2E)—and a 1.4 time increase in the IF1/ATP5a ratio that were abolished by siANT2 (Figure 4C–E). Although this latter value is probably underestimate because of the decrease in the total mitochondrial compartment (Figure S2E), it is still in agreement with our above-mentioned observation of a reduced F_1_F_0_ATPase activity in MFN2KD cells (Figure 4A). Like siANT2, loss of IF1 function prevented the decrease of F_1_F_0_ ATPase activity inMFN2 KD cells (Figure 4F).

As for mitochondria ATP content, the suppression of IF1 function in control cells had no detectable effect, whatever glycolysis was blocked or not by oxamate (Figure 4G). In MFN2 KD cells, the loss of IF1 had no significant effect on mitochondrial ATP content. Administration of oxamate in case of double IF1/MFN2 loss of function resulted in a dramatic reduction of the mitochondrial ATP content suggesting that the inhibition of F_1_F_o_ATPase by IF1 prevents the hydrolysis of the glycolytic ATP imported into mitochondria (Figure 4G).

**Figure 5:**
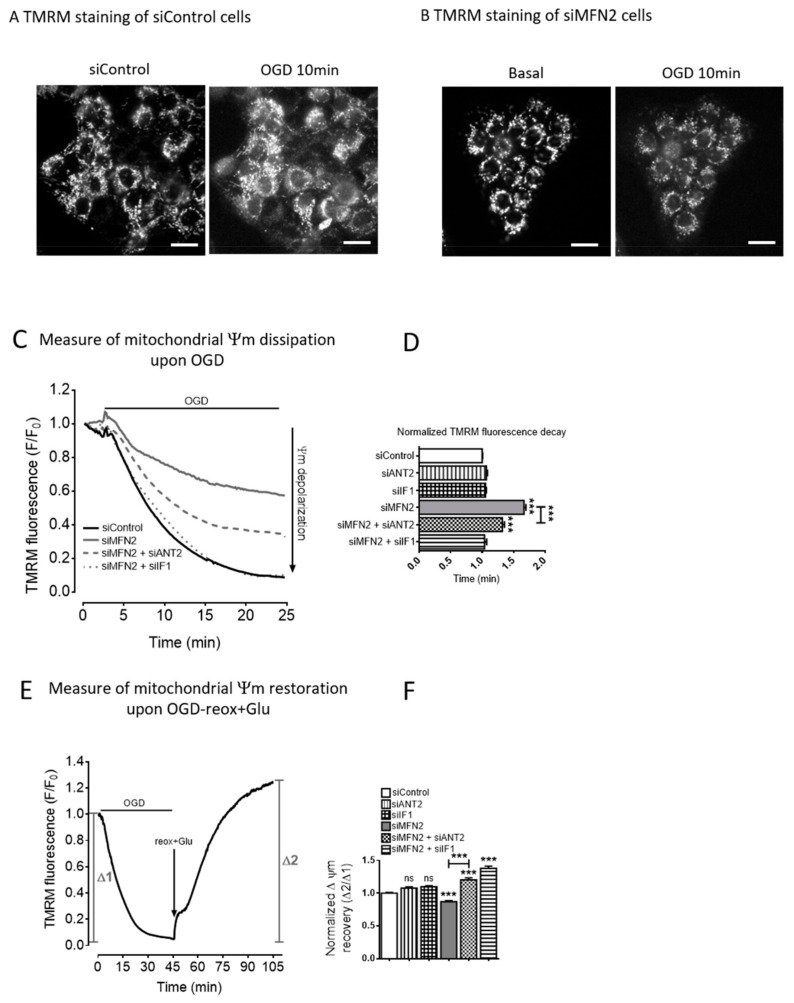
MFN2 loss upregulates ATPase inhibitory factor 1 preserving mitochondrial membrane potential. (**A**) Wide-field images of siControl H9C2-sv40 cells loaded with TMRM dye before and after a 10 min OGD (Scale bars, 20 μm). (**B**) Wide-field images of MFN2-KD H9C2-sv40 cells loaded with TMRM dye before and after an 10 min OGD (Scale bars, 20 μm). (**C**) Dissipation of mitochondrial membrane potential, Δψm, during OGD as measured by the variation in TMRM fluorescence in mitochondria of siControl (black), siMFN2 (grey), siMFN2 + siANT2 (dashed) and siMFN2 + siIF1 (dotted) transfected H9C2-sv40 cells (representative curves). (**D**) Δψm is characterized by the mean lifetime of an exponential decay fit on TMRM fluorescent time-lapse recordings. *n* = 276 (siControl), 383 (siANT2), 352 (siIF1), 289 (siMFN2), 192 (siMFN2 + siANT2), and 184 (siMFN2 + siIF1) cells. Mean lifetime of each condition has been normalized with the control’s mean lifetime of the experiment day. Data shown represent the mean ± SEM of 6 independent experiments. (**E**) Restoration of Δψm upon 60 min-reoxygenation with glucose after a 40 min-OGD as measured by the variation in TMRM fluorescence in mitochondria of H9C2-sv40 cells. (**F**) Values show mean ± SEMΔψm recovery after OGD and reoxygenation (reox + Glucose). (Δ2/Δ1) in4 independent experiments. H9C2-sv40 cells. *n* = 232 (siControl), 172 (siANT2), 284 (siIF1), 221 (siMFN2), 242 (siMFN2 + siANT2), 152 (siMFN2 + siIF1) cells. Δψm recovery of each condition has been normalized with the control’s Δψm recovery of the experiment day. *** *p* < 0.001.

We questioned whether the combination of the increased ATP import into mitochondria with the decreased hydrolysis by theF_1_F_0_ ATPase might contribute to prevent the mitochondria inner membrane depolarization during OGD. As shown in Figure 5, CTL cells displayed a major drop in the Δψm, and the suppression of either IF1 or ANT2 alone did not modified the Δψm mean lifetime during OGD (Figure 5A–D). MFN2KD cells underwent a lesser IMM depolarization than control cells (Figure 5C). As expected, the loss of IF1 function fully reversed this pattern during OGD. Importantly, the loss of ANT2 function partly prevented the protection against the IMM depolarization in the MFN2-KD cells.

We next assessed how ANT2 and IF1 participate in the restoration of Δψm at the onset of reperfusion (Figure 5E,F). In control cells, there was a marked IMM hyperpolarization upon oxygen and glucose restoration with Δψm averaging 1.360 ± 0.692 at one hour (Figure 5F). The suppression of either siIF1 or siANT2 alone did not modified the Δψm recovery after reoxygenation with glucose. MFN2 KD cells displayed a significantly attenuated IMM hyperpolarization at the onset of reoxygenation with glucose (Figure 5F). ANT2 and IF1 knock-down prevented this limited hyperpolarization in MFN2 KD cells and even induced a greater hyperpolarization than control cells (Figure 5F). These data suggest that both ANT2 and IF1 limit IMM hyperpolarization following OGD in MFN2 KD cells.

**Figure 6:**
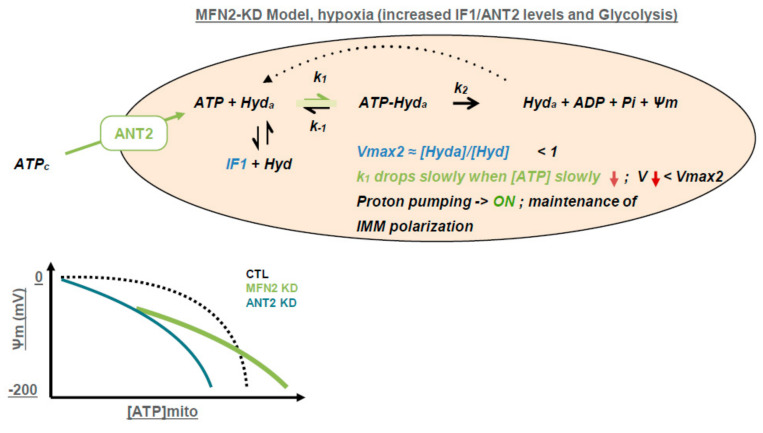
ANT2 and inhibitory factor 1 (IF1) mediate the modulation of mitochondria bioenergetics. Scheme summarizing the organization of cytosolic ATP import in mitochondria and IF-1 activity on the biochemical reaction of ATP hydrolysis and maintenance of Δψm in mitochondria of MFN2-KD cells. In the inset, curves represent a rough estimate of the relation between inner mitochondrial membrane (IMM) potential (ψm) and ATP concentration in mitochondria ([ATP]_mito_) in 3 cell populations transfected either with siCTL, siMFN2, or siANT2. This graph schematizes the effects of MFN2 KD shifting mitochondrial ATP concentration towards higher values and decreasing, in parallel, the maximal consumption rate of F_1_F_0_ ATP hydrolase via an increase in IF-1 expression. Cells knocked down for ANT2 show impaired ATP import which result in a shift of ATP concentration in mitochondria towards lower values.

As summarized in Figure 6, our results demonstrate that in cells knocked down for MFN2, IMM depolarization and repolarization during hypoxia–reoxygenation are modulated by F_1_F_0_ ATPase activity which depends on a minimum of two ordered reactions finely-tuned in MFN2 KD cells: (1) The ATP availability through the ANT2-mediated import into mitochondria, and (2) the F_1_F_0_ATPase reaction rate which is modulated by IF1. Subsequently, the prolonged proton pumping activity of the F_1_F_0_ATPase prevents the total dissipation of mitochondrial membrane potential.

We eventually investigated whether this metabolic shift could contribute to the protection against ischemia–reperfusion injury in MFN2 KD cells.

### 2.5. Import of Cytosolic ATP by Mitochondria Confers Resistance to Hypoxia

Opening of the mitochondrial permeability transition pore (PTP) plays a crucial role in cell death following a prolonged ischemia–reperfusion insult [1]. We assessed the mitochondrial Ca^2+^ retention capacity as measured by the calcein/cobalt method to evaluate the kinetic of PTP opening, as previously described [34].

**Figure 7:**
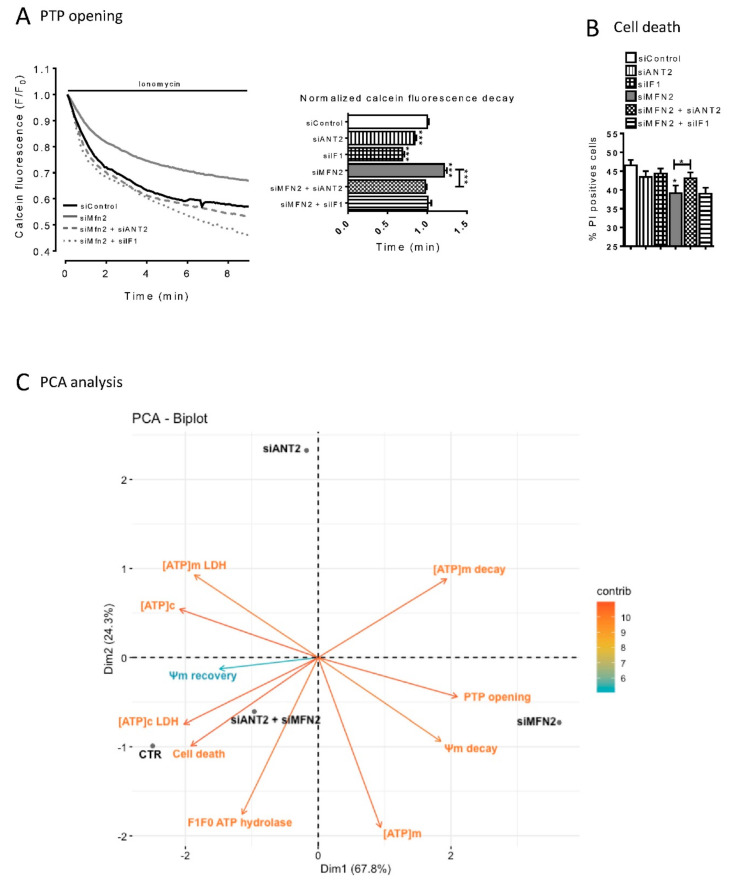
MFN2 loss triggers a metabolic reprogramming which confers resistance to hypoxic injury. (**A**) Calcein/cobalt experiment assesses the kinetic of PTP opening after treatment with ionomycin in siControl, siANT2, siIF1, siMFN2, and siMFN2 + siANT2 H9C2-sv40 cells. (left inset) Decay time of the calcein fluorescence decreases upon ionomycin treatment (right inset) *n* = 756, 110, 362,219, 282 and 365cells for siControl, siANT2, siIF1, siMFN2, siMFN2 + siANT2, and siMFN2 + siIF1 H9C2-sv40 cells, respectively. Data shown represent the mean ± SEM of 5 independent experiments. (**B**) Percentage of propidium iodide positive cells measured by FACS after 4 h OGD and 2 h reoxygenation in siControl, siANT2, siIF1, siMFN2, siMFN2 + siANT2, and siMFN2 + siIF1 H9C2-sv40 cells. Data shown represent the mean ± SEM of 9 independent experiments * *p* < 0.05; *** *p* < 0.001. (**C**) Principal component analysis shows the first and second principle components of the multi-variate scattering of 4 experimental conditions: Control (siCTL), MFN2 KD cells (siMFN2), ANT2 KD cells (siA) and MFN2/ANT2 KD cells (siAM) by the variance of mean values of 10 experimental variables: Steady-state cytosolic ATP concentration ([ATP]c), steady-state mitochondrial ATP concentration ([ATP]m), anaerobic glycolysis-dependent ATP level in cytosol ([ATP]c-LDH), mitochondrial ATP originated from cytosolic anaerobic glycolysis ([ATP]m-LDH), mPTP opening, cell death, ATP hydrolase activity during OGD (F1F0 ATP hydrolase), lifetime of the drop in mitochondrial ATP concentration during OGD ([ATP]m decay), lifetime of the drop in IMM potential during OGD (ψm decay) and the recovery of IMM potential after OGD-reoxygenation (ψm recovery). This shows that, regarding the 10 experimental variables, the phenotypes of cells knock-down for both ANT2 and MFN2 are similar to control cells. Conversely to control cells, single knocked-down cells either for MFN2 or for ANT2 display a dispersion along PC1 and PC2, respectively, demonstrating that they do not share an identical phenotype.

The calcein fluorescence decay time was significantly higher in MFN2-KD than in CTL cells (1.21 ± 0.04 min and 0.99 ± 0.02, respectively) suggesting an increased resistance to calcium-induced PTP opening (Figure 7A). Loss of function of ANT2 and IF1in control cells led to an increase in the rate of PTP opening (Figure 7A). In MFN2-KD cells, loss of function of ANT2 and IF1 also increased the rate of PTP opening suggesting that both players contribute to prevention of PTP opening in these cells.

Cell death was measured after a 4-h OGD followed by 2 h of reoxygenation. Flow cytometry measurements showed that loss of MFN2 function limited OGD-induced cell death (Figure 7B). Apoptosis and necrosis assay using FACS (Caspase and Propidium iodide) revealed that 90% of the cell death type induced by our OGD-reoxygenation protocol is necrosis (Figure S5B).

In CTL cells, the loss of either siIF1or siANT2 did not significantly affect cell death after OGD, indicating that neither of the two proteins alone is sufficient for protection against hypoxia–reoxygenation injury (Figure 7B). In contrast, the invalidation of ANT2, but not that of IF1, significantly attenuated the protection afforded by loss of MFN2 function (Figure 7B). In other words, both ANT2 and IF1 modulate Δψm and PTP opening but only ANT2 plays a significant role in cell death in MFN2 KD cells, suggesting that this metabolic switch might *per se* protect against hypoxia–reoxygenation injury.

### 2.6. ANT2 and IF1 Mediate the Modulation of Mitochondria Bioenergetics and Tolerance to Hypoxia-Reoxygenation in MFN2 KD Cells

Taking in account that molecular mechanisms are organized non-linearly, multivariate analysis like principal component analysis (PCA) can be used to question the contribution of values from different experiments in the proximity or dissimilarity of behavior of the different biological conditions. As shown in Figure 7C, PCA spreads four biological conditions: Control cells, MFN2-KD cells, ANT2-KD cells, and dual MFN2+ANT2-KD cells along the two principal components in function of the variance of each of the ten experimental measures.

A strong divergence in the phenotype of control cells and MFN2 KD cells is displayed by their scattering along the first PC (67.8% of variance) and is mostly related to modifications in the glycolysis/OXPHOS proportion (assessed by ATP production in with or without oxamate: A lactate dehydrogenase inhibitor), the lifetime of ATP content decrease during OGD and Δψm during OGD, PTP opening and cell death and steady-state ATP content in mitochondria. In contrast, the lone suppression of ANT2 in control cells spreads orthogonally, along the second PC (24.3% of variable variance), and is mostly related F_1_F_0_ ATP hydrolase activity and steady-state mitochondrial ATP concentration. Strikingly, MFN2+ANT2-KD and ANT2-KD cells spread orthogonally to each other—indicating a major difference in the phenotype reported by the ten experimental measures. Consequently, no direct comparison on the role of ANT2 suppression should be drawn by comparing its effect in control and MFN2 KD cells for a single experimental variable. Finally, suppression of ANT2 in MFN2 KD cells almost fully reverse their protective bioenergetic phenotype.

The suppression of IF1 in MFN2 KD was insufficient to reverse fully the MFN2 KD phenotype (Figure S5A). Since ANT2 intervene “upstream” of IF1 in the biochemical process (Figure 6), this analysis supports the fact that ATP import in mitochondria did not only improve F_1_F_0_ATP hydrolase activity synergistically to IF1, but also likely enhanced other ATP-dependent activities.

## 3. Discussion

Our study shows that the protection against hypoxia–reoxygenation injury afforded by the loss of MFN2 function is partly due adaptations leading to metabolic shift towards glycolysis and overexpression of ANT2 working as an importer of cytosolic ATP into mitochondria. The concurrent modulation of the F1FoATPase by IF1 is a secondary component of the protective effect triggered by MFN2 deletion.

### 3.1. A Metabolic Shift to Improve Cell Tolerance to Hypoxia–Reoxygenation

Studies have previously reported that cells lacking MFN2 display glucose metabolism impairments even though they did not report major change in cell bioenergetics. To our knowledge no difference in ATP content has so far been reported. As reviewed by E. Schrepfer and L.Scorrano, in different cell models, MFN2 depletion leads to a reduction of the mitochondrial membrane potential, of cellular oxygen consumption, of mitochondrial co-enzyme Q levels and of the expression of OXPHOS complexes I, II, III, and V [16].

Reportedly, total cellular ATP level was unchanged in MFN2 KD or KO cell models [35,36]. Although we detected no difference in cytosolic ATP content before or after hypoxia, mitochondrial ATP content was increased in MFN2 KD cells under both normoxia and hypoxia. Because cell lines mainly display a glycolytic phenotype, their mitochondrial respiration is low and small differences between control and MFN2 KD cells might have been hidden. As previously proposed [16], cells lacking MFN2 might compensate the decrease in the OXPHOS-mediated ATP synthesis by increasing glucose uptake, shifting to anaerobic glycolysis and generate ATP according to the Warburg model [37]. Using the fluorescent biosensor for cytosolic NADH-NAD^+^ we showed that cells with a reduced MFN2 expression, do rely on anaerobic glycolysis to produce ATP. We showed the non-specificity of this Warburg-like adaptation by showing it in three cell types of different tissues and organisms.

Reports suggest that cancer cells express ANT2 in order to transport cytosolic ATP into mitochondria [30]. This process appears to drive the clonal selection occurring in the tumor core [38]. However, it is so far unclear whether this metabolic shift might occur in a non-cancer condition. The participation of ANT2 to ATP transport across the mitochondrial membrane has been challenged [39], mainly based on the only measurement of the mitochondrial membrane potential. The best way to determine whether ANT2 is really able to transport ATP in/out mitochondria is to measure changes in ATP concentration on each side of the inner mitochondrial membrane in presence of an ANT2 inhibitor or in an ANT2 knock-down/out condition. Using this approach, we demonstrated that ANT2 works as an ATP exporter in control cells and as an importer when MFN2 function is loss. However, the mechanism of this reversible ATP transport by ANT2 remains to be determined.

### 3.2. Respective Role of ANT2 and IF1 to Maintain Mitochondrial ATP

We demonstrated that loosening the tight coupling between ER and mitochondria via loss of MFN2 function decreases the OXPHOS metabolism and increases ANT2 expression that, in turn, runs the import of cytosolic ATP in the mitochondria. This allowed to prevent the polarization of the inner mitochondrial membrane under normoxia, restrained its depolarization during hypoxia and its hyperpolarization at the onset of reperfusion, and finally, it attenuated cell death after a prolonged hypoxia–reoxygenation. Suppression of ANT2 prevents these events confirming the requirement for ATP import into mitochondria likely to fuel F_1_F_o_ATP hydrolase during hypoxia. We found a partial decoupling between ANT2-mediated ATP import and the reduced activity of F_1_F_o_ATP hydrolase. This latter was mainly due to the increased expression of theF_1_F_o_ATP hydrolase inhibitor IF1. This partial uncoupling between ATP import and ATP hydrolase is most likely responsible for the increased content of ATP in mitochondria of MFN2 KD cells and provides the best equilibrium between rates of ATP consumption and proton pumping during hypoxia. Indeed, in control cells, the absence of ANT2-mediated ATP import and the suppression of IF1 leads to a burst of F_1_F_o_ATP hydrolase activity during hypoxia that conduct to a quick depolarization of IMM and cell death. As represented on the Figure 6, as long as ANT2 transport rate is greater than ATP hydrolase + other ATP-consuming process, the IMM potential is maintained. However, because IF-1 expression level and activity are defining the percentage of functional ATP hydrolase, it may, secondarily to ANT2, help to limit the maximal rate of ATP hydrolase reaction.

### 3.3. Mitochondrial ATP, Inner Membrane Polarization, and Cell Death

Although both ANT2 and IF-1 are essential for the control of IMM polarization and the Ca^2+^-induced PTP opening, their role in cell protection against ischemia–reperfusion remains poorly understood. We found that IF1 suppression reversed the partial depolarization of the IMM at reperfusion in MFN2 KD cells and even caused a significant hyperpolarization (Figure 5D). This confirms that IF1 is a key component of the MFN2-mediated shift in IMM polarization response to OGD and reperfusion. The same analysis also depicts a negative correlation between mitochondrial ATP content and cell death (Figure 7C). The combined IF1 and MFN2 loss of function increased the mitochondrial ATP content while protecting against cells death. Conversely to IF1 and MFN2, the combined ANT2 and MFN2 loss of function decreased mitochondrial ATP content and increased cell death following OGD-reperfusion. This analysis suggests that cytosolic ATP import in mitochondria is required for the protection afforded by MFN2 KD against OGD-reperfusion, and ATP degradation by F_1_F_0_ATP hydrolase is probably not the unique mechanism involved in the protection.

Interestingly, Ca^2+^ induced PTP opening did not predict cell death upon hypoxia–reoxygenation in our study (Figure 7A,B). Although IF-1 suppression reversed the MFN2 KD-mediated inhibition of PTP opening, it did not prevent the protective effect of MFN2 KD on cell death after hypoxia–reoxygenation. This suggests that even though the Ca^2+^-induction of PTP is restored to the control levels in MFN2/IF1 KD cells, the open probability of the PTP during hypoxia–reoxygenation might be lowered in the latter as well as in MFN2 KD cells. Since we observed that the rate of ψm loss during OGD was comparable in control and IF-1/MFN2 KD cells, it is likely that ATP hydrolase activity is restored to the control level in the latter cells. This suggests that while cytosolic ATP is shuttled towards mitochondria, ψm loss is not sufficient to trigger cell death.

**Figure 8:**
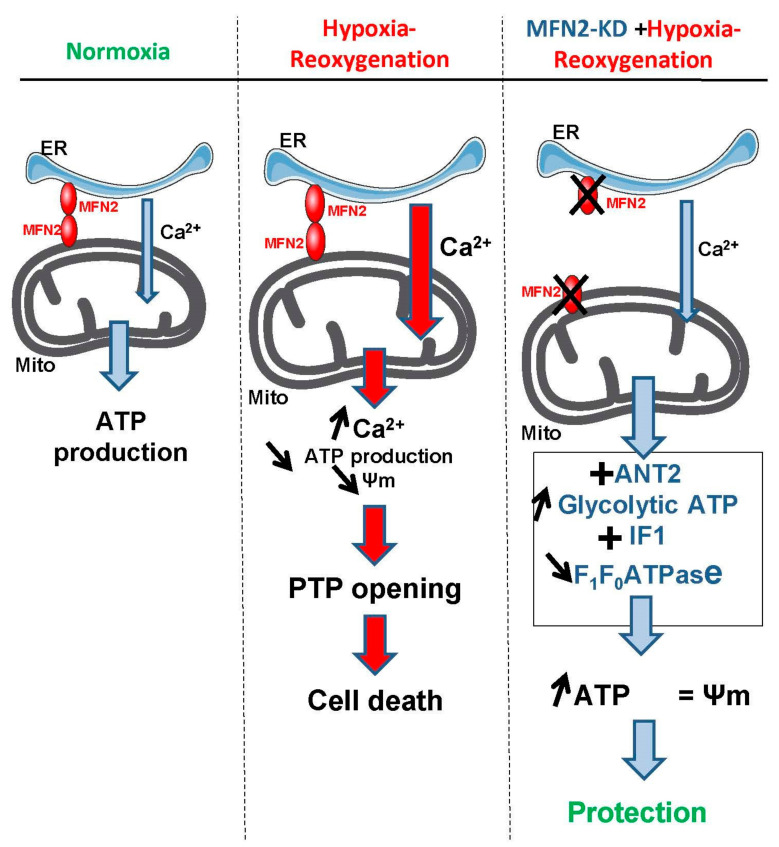
ANT2-mediated ATP import into mitochondria protects against cardiac hypoxia lethal injury. Ischemia-reperfusion injury triggers an endoplasmic reticulum (ER) Ca^2+^ transfer to mitochondria (Mito), leading to membrane potential imbalance and opening of the permeability transition pore (PTP). Loss of MFN2 disrupts ER-mitochondria contact sites, lessened ER-to-mitochondria Ca^2+^ transfer and caused a metabolic reprogramming (Warburg shift) of the cell bioenergetics. The increased import of cytosolic ATP into mitochondria via ANT2 and the fine-tuning of F_1_F_0_ATP hydrolase by IF1 overexpression allowed a better tolerance to hypoxia by mean of the prevention of the collapse of the mitochondrial membrane potential associated to a delayed opening of the permeability transition pore and a higher steady-state ATP content during hypoxia associated with a greater cell survival.

In conclusion, our study brings new insights into the mechanism of cell protection against hypoxia–reoxygenation. It is commonly accepted that accumulation of Ca^2+^ into mitochondria plays a major role in cell death following a prolonged hypoxia–reoxygenation insult by directly triggering PTP opening. Limiting Ca^2+^ overload by untethering ER (e.g., via loss of MFN2 function) and mitochondria would therefore appear as a sound therapeutic option. We here showed that this ER-mitochondria uncoupling, on top of limiting mitochondria Ca^2+^ overload, induced a major metabolic adaptation consisting primarily in the overexpression of ANT2 to import glycolytic ATP and of IF1 to limit ATP degradation by the F1FoATPase. The subsequent preservation of mitochondria ATP and maintenance of inner mitochondria membrane polarization per se significantly contributed to prevent cell death induced by a sustained hypoxia–reoxygenation insult (Figure 8).

## Supplementary Materials

The following are available online at https://www.mdpi.com/2073-4409/9/12/2542/s1, Figure S1: siRNA/shRNA validation on different cell types (H9C2-sv40, MEFs, AML12 cells). Figure S2: The Calcium/ATP paradox in MFN2 KD cells. Figure S3: MFN2 loss of function induces a glycolytic phenotype. Figure S4: Validation of protein silencing. Figure S5: Related to Figure 7. PCA analysis. Figure S6: pH sensitivity of cameleon fluorescent protein.
