# Addendum: Gouriou et al. ANT2-Mediated ATP Import into Mitochondria Protects against Hypoxia Lethal Injury. *Cells* 2020, *9*, 2542

**DOI:** 10.3390/cells10092171

**Published:** 2021-08-24

**Authors:** Yves Gouriou, Muhammad Rizwan Alam, Zeina Harhous, Claire Crola Da Silva, Delphine Baetz, Sally Badawi, Etienne Lefai, Jennifer Rieusset, Annie Durand, Rania Harisseh, Abdallah Gharib, Michel Ovize, Gabriel Bidaux

**Affiliations:** 1 Univ-Lyon, CarMeN Laboratory, INSERM 1060, INRA 1397, Université Claude Bernard Lyon1, INSA Lyon, Oullins, France, IHU OPERA, Groupement Hospitalier EST, Bâtiment B13, 59 boulevard Pinel, F-69500 Bron, France; rizwan805@gmail.com (M.R.A.); zeina.harhous@gmail.com (Z.H.); claire.crola-da-silva@univ-lyon1.fr (C.C.D.S.); delphine.baetz@univ-lyon1.fr (D.B.); sally_b86@hotmail.com (S.B.); Etienne.Lefai@inra.fr (E.L.); jennifer.rieusset@univ-lyon1.fr (J.R.); annie.durand@univ-lyon1.fr (A.D.); rania.harisseh@gmail.com (R.H.); abdallah.gharib@univ-lyon1.fr (A.G.); michel.ovize@gmail.com (M.O.); 2 Department of Biochemistry, Quaid-i-Azam University, Islamabad 45320, Pakistan; 3 Gilbert and Rose-Marie Chagoury, School of Medicine, Lebanese American University, Byblos 4M8F+8X, Lebanon

The authors and the *Cells* Editorial Office would like to add the section “Materials and Methods”, which was missing in the original version [1]. We apologize for any inconvenience caused and state that the scientific conclusions are unaffected.

## 4. Materials and Methods

### 4.1. Experimental Model and Subject Details

#### 4.1.1. pH Sensitivity of the YFP-Based Biosensor in OGD Experiments

YFP-based FRET biosensors are known to be pH sensitive, which makes them artifact prone. Indeed, the original cameleons suffered from significant pH sensitivity, which has been improved in order to decrease the pKa of the acceptor YFP. Two mutations within EYFP (V68L and Q69K) were used to decrease its pKa to 6.1 [20–22]. The situation becomes even worse in the specific case of the acidosis occurring during hypoxia. However, this matter has rarely been assessed in the literature. In this study, we measured the ATP-specific response of an ATeam sensor in MFN2-KD cells during ODG by quantifying its pH-sensitive drop in the FRET signal by means of an engineered cameleon fluorescent biosensor deprived of the ligand domain, “null sensor” (Figure S6). We provided a calibration of this null sensor in different pH solutions from pH 8 to pH 5.3 and estimated the cytosolic pH of our cells as 6.7 during OGD. In addition, our experiments clearly demonstrated that less than 20% of the ATeam variation was subject to pH sensitivity. Although, this result prevents a precise quantification of ATP variation, it does not prevent a qualitative analysis of the ATP level through the OGD-reperfusion sequence.

#### 4.1.2. Transient MFN2 KD vs. KO

Many discrepancies have been published in the MFN2 field depending on the genetic perturbation, the model, and the techniques used. Cardiac myocytes (KO model), cultured neonatal rat myocytes (KD model), MEF cells (KO model), neurons (KO and KD model), and Sh-SY5Y cells (KD model) have been used since 2011 [2,6,11,23–26]. For example, in a KO model of cardiac myocytes the authors observed resistance to permeability transition opening and less tunnel positive dead cells after ischemia-reperfusion (IR), and the opposite results in a cultured neonatal rat myocytes MFN2 KD model. They proposed that the action of MFN2 on PTP is likely to be cell type specific, depending on the extent of cellular differentiation [25]. In our laboratory, we observed less cell death after IR in a KD model of rat cardiomyoblast cell line [27]. In 2015, Filadi et al. measured a 50% reduction of MCU expression in a MFN2 KO model of MEF cells, whereas they recorded no difference in MCU expression in a MFN2 KD model of SHSY cells [11].

The major drawback in KO models lies in the plasticity of the genome, which enables adaptation/compensation at the gene level, towards a novel equilibrium of the molecular network being reached. Although it is always tempting to infer that a modified phenotype is directly caused by the suppression of the target gene, this should not be assumed so casually without controlling the state of the whole molecular network. Regarding the controversies in the literature on MFN2 models, and because we aimed at measuring the early molecular adaptations to MFN2 loss and their effect on cell in hypoxia-reoxygenation, we decided to use a KD model of MFN2. As reported in this study, we observed strong phenotypic modifications only two days after silencing of MFN2, which confirmed our intuition that transient KD can be a good model to probe the early events induced by changes in the expression of a target gene.

### 4.2. Cell Culture

Immortalized rat cardiomyoblasts cell line H9C2-sv40 cells were grown in DMEM 4.5 g/L glucose, l-glutamine containing 10% fetal bovine serum, and 100 units/mL of penicillin and streptomycin. Cells were cultured in 95% air and 5% CO_2_ at 37 °C and subcultured every 5 days at a 1:10 dilution. Cells were plated either on coverslips or on plates (Ibidi GmbH Inc., Gräfelfing, Germany and MatTek Corp., Ashland, MA, USA) and grown for 24 h before transfection with siRNA and/or fluorescent proteins using Dharmafect (GE Dharmacon, Lafayette, CO, USA) duo reagent. Experiments were performed 48 h after transfection. Mitofusin-2 (MFN2) silencing was achieved with 100 nM of siRNA (Qiagen, no.SI01907395, Hilden, Germany) (Figure S1A,B). The same procedure was applied for siRNA, ANT2, and IF1 (Eurogentec, Liège, Belgium) (Figure S4C,D). In parallel, controls were transfected with 100 nM of scramble siRNA (Qiagen, no 1027281). The knock-down efficiency was verified by Western-blotting and quantitative PCR (Figure S1C,D). AML12 (alpha mouse liver 12) cell line was grown in DMEM F12 (L0090-500) containing 10% fetal bovine serum, 100 units/mL of penicillin and streptomycin, Dexomethasone (40 ng/mL, Sigma D4902-100MG, St. Louis, MO, USA), insulin solution human (5 µg/mL, Sigma I9278-5ML), Transferrine (5 µg/mL, Sigma T8158-100MG), and sodium elenite: (5 ng/mL, Sigma S5261-25G). Cells were cultured in 95% air and 5% CO_2_ at 37 °C. Mouse embryonic fibroblast (MEF) cell line was grown in DMEM 4.5 g/L glucose, l-glutamine containing 10% fetal bovine serum, 10 mM pyruvate, and 100 units/mL of penicillin and streptomycin. Cells were cultured in 95% air and 5% CO_2_ at 37 °C.

### 4.3. Adenovirus Infection

The infection of cells with adenovirus-mCherry-shRNA (scramble or MFN2) at 10 MOI was performed 6 h after plating of the cells (MEF or AML12 cells) and followed by transfection with fluorescent protein 24 h after plating. Knock-down efficiency was verified by Western-blotting. Adenovirus constructs were obtained from Vector Biolabs, Malvern, PA, USA (Backbone: Adenoviral-Type 5(dE1/E3), promoter U6, mouse MFN2 shRNA, tag: mCherry driven by CMV promoter) (Figure S1C,D).

### 4.4. Cell Lysis and Western Blotting

Cell lysates were obtained by treating the cell monolayer with RIPA buffer supplemented with 1 mM Na_3_VO_4_, 1 mM DTT, 20 mM NAF, 5mM EDTA, and a cocktail of proteases inhibitor. Total protein concentration was determined using the bicinchoninic acid method (BCA, Interchim, Montluçon, France), and 25 µg of protein from each sample was loaded on 12% sodium dodecyl sulfate polyacrylamide gel (SDS-PAGE). Migration was performed for 15 min at 90 V, followed by 60 min at 130 V. Proteins were then blotted on a polyvinylidene difluoride (PVDF) membrane by electro transfer (Trans-Blot Turbo Transfer, Bio-Rad, Hercules, CA, USA). The PVDF membrane was incubated at room temperature for 1 h with 5% milk in PBS for blocking and then incubated overnight at 4 °C in the same buffer with the primary antibody. Secondary horseradish peroxidase (HRP) coupled antibodies, an ECL (entry-level peroxidase substrate for enhanced chemiluminescence) plus kit, and Western Blotting detection system from GE Healthcare were then used to reveal antigen–antibody complexes. The band intensity was determined using ImageLab software (Bio-Rad).

### 4.5. Oxygen-Glucose Deprivation (OGD) Experiments

Cells were washed twice in a HEPES-buffered solution containing 140 mM NaCl, 5 mM KCl, 1 mM MgCl_2_, 2 mM CaCl_2_, 10 mM HEPES, 2 mM Na_2_S_2_O_4_ (pH 7.4) with NaOH at 37 °C, placed into a specifically manufactured bio-incubator (NewBrunswik, an Eppendorf company, Galaxy 48R), and connected with a 100% N_2_bottle at 37 °C. Oxygen levels were monitored at 0.5% O_2_ by the system. After the OGD period, cells were washed twice with a HEPES-buffered solution containing 10 mM glucose and cultured in complete medium for 4 h in an incubator with 95% air and 5% CO_2_ at 37 °C. For the OGD experiments, on the microscope an Okolab system with a specific hypoxic chamber was used which controlled the environment (temperature, humidity, oxygen levels).

### 4.6. Wide-Field Microscopy for ATP, NADH/NAD^+^, and Ca^2+^ Live Cell Imaging

Cells were imaged on an Leica epifluorescence microscope using 40× objective. The [Ca^2+^] in cytosol, mitochondria, and ER were measured with D3cpv, 4mtD3cpv, and erGAP1, respectively. [ATP] in cytosol and mitochondria was measured with ATeam-cyto and ATeam-mito. Of note, the Ateam dynamic range was characterized between 0.5 and 5 mM [16]. With a steady-state ATP concentration at the mM concentration in cells, the Ateam sensor reached its insensitivity range in basal conditions; therefore, the increases in basal ATP level are prone to underestimation (Figure 1B).

Cytosolic NADH/NAD^+^ ratio was measured with Peredox [15]. Fluorescence ratios were calculated in MetaFluor 6.3 (Universal Imaging, Bedford Hills, NY, USA) and analyzed in Origin Pro (OriginLab, Northampton, MA, USA) + GraphPad Prism 4 (GraphPad, San Diego, CA, USA).

The proportion of aerobic-dependent and anaerobic-dependent ATP synthesis within the dynamic range of the Ateam sensors was calculated as following:

The minimal (*F_YFP_/F_CFP_*) fluorescence ratio value, (*F_YFP_/F_CFP_*)*_min_*, corresponding to the minimal sensitivity of Ateam probes to ATP was determined in cells incubated with oxygen/glucose-deprivation in the presence of 2 µM oligomycin A. At a constant illumination power and exposure time set on the microscope, the (*F_YFP_/F_CFP_*)*_min_* value was 3858 ± 0.029 (*n* = 409). (*F_YFP_/F_CFP_*)*_min_* was first subtracted from the (*F_YFP_/F_CFP_*) fluorescence ratio values at all times to obtain the corrected (*F_YFP_/F_CFP_*) values. Corrected (*F_YFP_/F_CFP_*) values measured in presence of an inhibitor were normalized by the corrected (*F_YFP_/F_CFP_*) values measured in the absence of this inhibitor (control). Finally, this ratio was subtracted by (1). The whole formula is:

(1) $$\;\mathrm{Treatment-dependent}\;\mathrm{ATP}\;\mathrm{synthesis}=1-\frac{{\left(F_{YFP}/F_{CFP}\right)}_{inhibitor}-{\left(F_{YFP}/F_{CFP}\right)}_{min}}{{\left(F_{YFP}/F_{CFP}\right)}_{control}-{\left(F_{YFP}/F_{CFP}\right)}_{min}}$$

Unlike Ateam and Ca-sensing probes, the Peredox probe does not utilize FRET measurements but works by the principal of direct quantification of fluorescence intensity. As the direct quantification of fluorescence intensity is biased, we applied the method proposed by [15]. In this procedure, the steady-state green/red fluorescence ratio is normalized by the that measured after a 10 mM pyruvate treatment on cells. This high dose of pyruvate enhances the lactate dehydrogenase activity, which consumes NADH at a higher rate than it can be regenerated. Normalization of the (*F_green_/F_red_*)*_basal_* ratio by this minimum Peredox signal, (*F_green_/F_red_*)*_pyruvate_*, prevents artefacts related to variations in the probe concentration.

(2) $$Normalized\;\frac{NADH}{NAD^{+}}ratio=\frac{{\left(F_{green}/F_{red}\right)}_{basal}}{{\left(F_{green}/F_{red}\right)}_{pyruvate}}$$

### 4.7. Calcein Cobalt Protocol

Kinetics of mPTP opening was measured in live cells by means of the well-established calcein-cobalt assay. Briefly, cells were loaded with 1 µM calcein-AM in a Ca^2+^-containing physiological solution (CCB) for 15 min at room temperature. Cells were washed twice and incubated for another 30 min with 2 mM cobalt chloride and 200 µM sulfinpyrazon in CCB. Using a Leica wide-field microscopy system, cells were imaged every 5 s under resting conditions for 1–2 min before application of 1 µM ionomycin, which triggers the mPTP opening. Calcein-AM was excited using a GFP excitation filter and emission was performed with a FITC filter. A decrease in mitochondrial calcein fluorescence reflected the opening of mPTP. For data analysis, the background was subtracted and curves were normalized with the basal fluorescence.

### 4.8. FACS Analysis of Cellular Viability and Mitochondrial Membrane Potential

FACS analyses of propidium iodide positive cells were performed 4 h after OGD. Cells were harvested with Accutase (GE Healthcare, L11-007), centrifuged, and resuspended in complete medium. PI at a 1 µg/mL final quantity was added to perform FACS analysis by Fortessa X-20 (BD Biosciences, San Jose, CA, USA) (λex 530 nm, λem 620 nm). FACS analysis of mitochondrial membrane potential was performed with two different dyes. TMRM at 20 nM (Fortessa X20::λex 561, λem 580 nm) and DILC1(5) at 20 nM (Fortessa X20::λex 640, λem 658 nm). To obtain a minimum basal value for mitochondrial membrane potential with each dye, cells were incubated with 10 µM FCCP for 10 min. TMRM fluorescence was normalized with the minimum fluorescence intensity obtained after FCCP treatment.

### 4.9. RNA Extraction and Real-Time PCR

Two days after transfection, cells were harvested by trypsination and total RNA isolation was performed using Trizol technique, according to the manufacturer’s protocol, and reverse-transcribed using a High-capacity RNA-to-cDNA kit (Applied Biosystems, 4387406, Foster City, CA, USA). Real-time PCR was performed using the StepOnePlus real-time PCR system (Applied Biosystems) and Fast SYBR Green Master Mix (Applied Biosystems, 4385612) according to the manufacturer’s instructions. Technical triplicates were made for each condition. All primers used are shown in the Key Resource table.

### 4.10. Principal Component Analysis

Data were subjected to the multivariate statistical method of principal component analysis (PCA) implemented in *R language* version 3.3.3, freely available at https://cran.r-project.org/ (accessed on 2 August 2021). PCA is an unsupervised method that aims to reduce data dimensionality via identifying a smaller number of uncorrelated variables known as principal components (PC). The PC1 axis represents efficiently the dataset and accounts for the highest variation in the original data. However, the second PC, perpendicular to PC1, accounts for most of the remaining variances. In order to analyze and visualize the data, the *prcomp()*, *fviz_pca_ind()*, and *fviz_pca_var()* functions, supplied with the “*factoextra*”and “*ggplot2*” packages, were used, respectively.

### 4.11. Quantification and Statistical Analysis

The significance of differences between means was established using Student’s *t* test for unpaired samples (* *p* < 0.05; ** *p* < 0.01; *** *p* < 0.001) and a non-parametric Kruskal–Wallis test with Dunn’s post hoc test.
