# Supplementary material for: ANT2-Mediated ATP Import into Mitochondria Protects against Hypoxia Lethal Injury
Source: Cells. 2020 Nov 25;9(12):2542. doi: 10.3390/cells9122542 (PMC7760820; doi:10.3390/cells9122542)
Supplement: Supplementary file 1 [file cells-09-02542-s001.pdf]

Supplemental Information

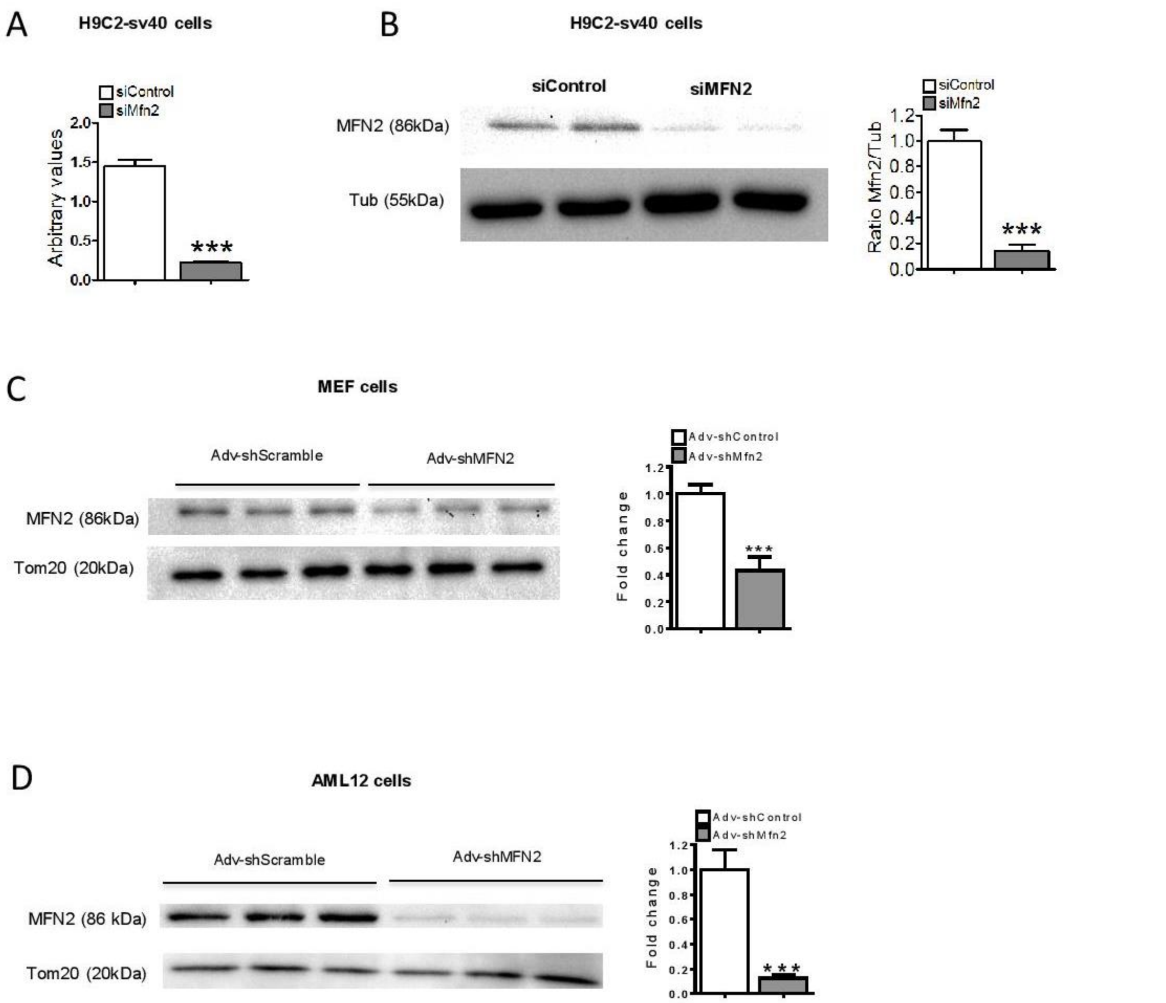

Figure S1: siRNA/shRNA validation on different cell types (H9C2-sv40, MEFs, AML12 cells).

(A)Histogram shows MFN2 mRNA expression level quantified by quantitative-PCR in control and MFN2-KD H9C2-sv40 cells. Data shown represent the mean ± SEM of 3 independent experiments. (B) Western-blot showing the expression ofMFN2 proteins in control and MFN2-KD H9C2-sv40 cells. MFN2 expression was normalized by the one of Tubulin. Data shown represent the mean ± SEM of 3 independent experiments. (C) Western-blot showing the expression ofMFN2 proteins in Adv-shScramble and Adv-shMFN2 MEF cells. MFN2 expression was normalized by the one of Tom20. Data shown represent the mean ± SEM of 3 independent experiments. (D) same as (C), in Adv-shScramble and Adv-shMFN2 AML12 cells. Data shown represent the mean ± SEM of 3 independent experiments.

Cells **2020**, *9*, 2542; doi:10.3390/cells9122542 www.mdpi.com/journal/cells

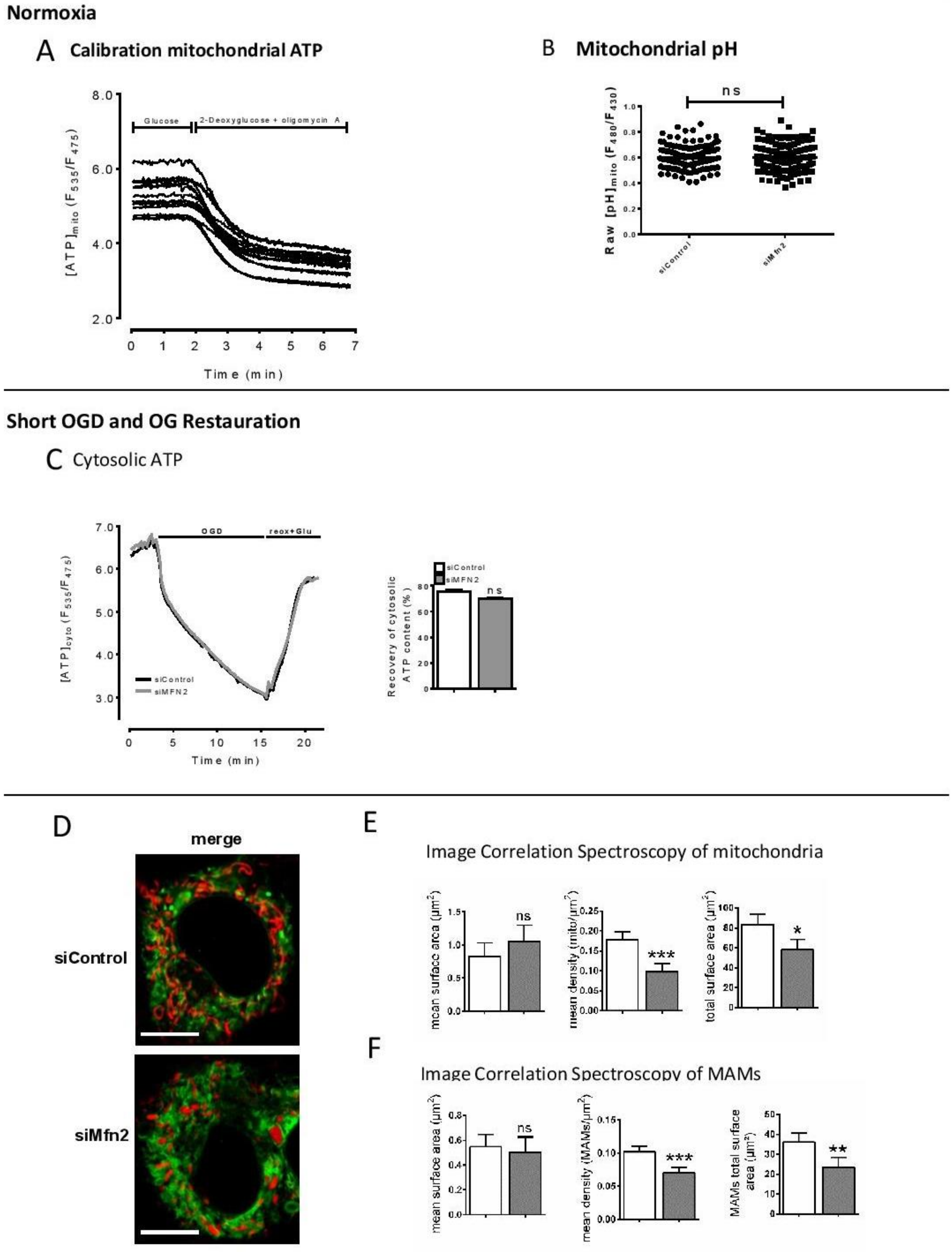

Figure S2: The Calcium/ATP paradox in MFN2 KD cells.

(A) Calibration of Mitochondrial ATP measurements with Ateam biosensor in H9C2-sv40 cells before (steady-state $[ATP]_{mito}$) and upon treatment with 2-Deoxyglucose and oligomycin A (minimal $[ATP]_{mito}$). Corrected steady-state mitochondrial ATP level ($Corr[ATP]_{mito}$) calculated as ratio of Ateam values before/after 2-Deoxyglucose and oligomycin A for each cell (right inset). *n* = 126, 139 cells for siControl and siMFN2 H9C2-sv40 cells, respectively. Data shown represent the mean ± SEM of 4 independent experiments. (B) Steady-state mitochondrial pH measurement ratio value in control and

MFN2-KD H9C2-sv40 cells. *n*= 145, 109 cells for siControl and siMFN2 H9C2-sv40 cells, respectively. Data shown represent the mean ± SEM of 3 independent experiments. (C) Cytosolic ATP level during a 15min-oxygen glucose deprivation (OGD) and a 5min-reoxygenation with glucose (reox+Glucose), in control (black) and MFN2-KD (grey) H9C2-sv40 cells. (right inset) Percentage of cytosolic ATP recovery is calculated as mitochondrial ATP recovery in control (white) and MFN2-KD (grey) H9C2-sv40 cells. *n*= 154, 149 cells for siControl and siMFN2 H9C2-sv40 cells, respectively. Data shown represent the mean ± SEM of 3 independent experiments. (D) Cells transfected with an ER calcium sensor (D1ER) and loaded with MitoTracker DeepRed. *n* = 59 cells for siControl and MFN2-KD H9C2-sv40 cells (Scale bars, 10 µm). (E) Image Correlation Spectroscopy (ICS) analysis of mitochondria. (left inset) mean surface of mitochondria (µm²). (middle inset) mean density of mitochondria (mitochondria/µm²) and (right inset) total mitochondrial surface (µm²). (*n* = 64, 59 cells for siControl and MFN2-KD (grey bar) H9C2-sv40 cells, respectively. (F) ICS analysis of ER-mitochondria interactions. (left inset) ER/mitochondria interactions surface (µm²). (middle inset) ER/mitochondria interactions density (mitochondria/µm²) and (right inset) total ER/mitochondria interactions surface (µm²). *n* = 63, 68 cells for siControl and MFN2-KD (grey bar) H9C2-sv40 cells, respectively. Results shown are the mean ± SEM of 4-5 independent experiments; ***P < 0.001.

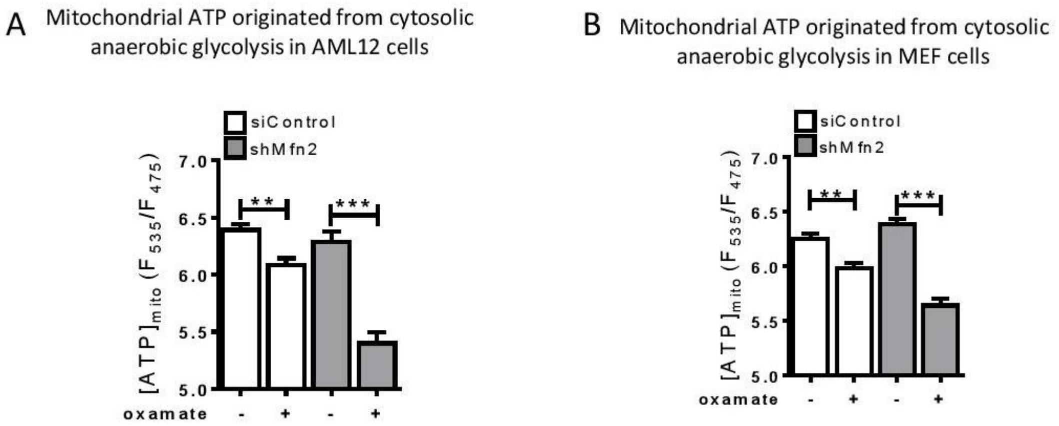

Figure S3*:* MFN2 loss of function induces a glycolytic phenotype

(A) and (B)Live measurement of steady-state mitochondrial ATP and after incubation with oxamate (20mM) in order to calculated the proportion of mitochondrial ATP originating from anaerobic glycolysis in AML12 and MEF cells, respectively. Control (white) ± oxamate and shMFN2 (grey)± oxamate AML12 cells: *n* = 237, 139, 130, 136 cells. Control (white) ± oxamate and shMFN2 (grey)± oxamate MEF cells: *n* = 186, 179, 214, 151 cells. Data shown represent the mean ± SEM of 4 independent experiments. **P< 0.01; ***P< 0.001.

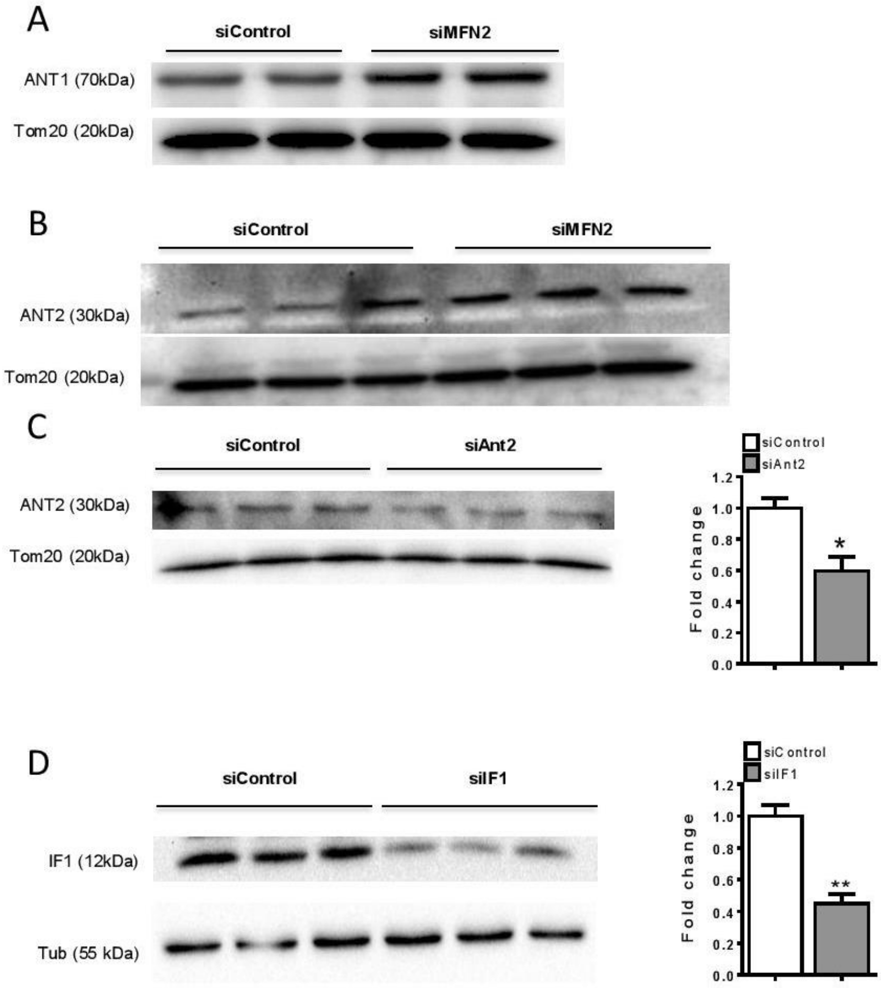

Figure S4: Validation of protein silencing.

(A) Western-blot of showing ANT1 expression in MFN2-KD H9C2-sv40 cells. The mitochondrial protein Tom20 is used as a control of a protein loading and for normalization of ANT1 expression as shown in the figure 3A. (B) same as (A) for ANT2 detection. Normalization of ANT2 expression level by tom20 is shown in the figure 3B. (C) Western-blot shows Ant2 protein level in control and Ant2-KD H9C2-sv40 cells. ANT2 was normalized to Tom20 protein. Data shown represent the mean ± SEM of 3 independent experiments. (D) Western-blot shows IF1 protein level in control and IF1-KD H9C2-sv40 cells. IF1 was normalized to Tubulin protein Data shown represent the mean ± SEM of 3 independent experiments. **P*< 0.05; ** *P*< 0.01; ****P*< 0.001.

A

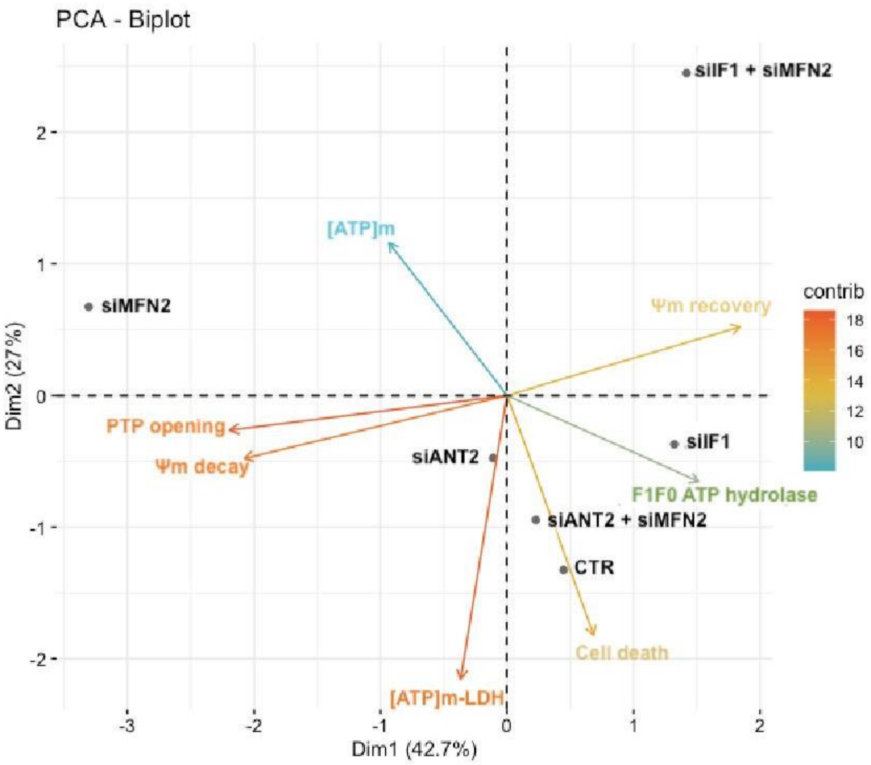

B

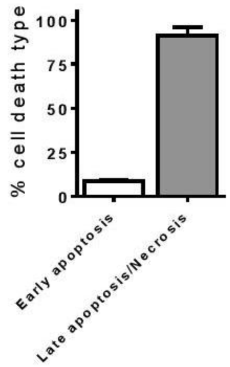

Figure S5*: Related to Figure 7.* PCA analysis

(A) Principal component analysis shows the first and second principle components of the multi-variate scattering of 6 experimental conditions: control (siCTL), MFN2 KD cells (siMFN2), IF1 KD cells (siIF1), MFN2/IF1 KD cells (siIF1+siMFN2), ANT2 KD cells (siA) and MFN2/ANT2 KD cells (siAM) by the variance of mean values of 7 experimental variables: steady-state mitochondrial ATP concentration ([ATP]m), mitochondrial ATP originated from cytosolic anaerobic glycolysis([ATP]m-LDH), PTP opening, cell death, ATP hydrolase activity during OGD (F1F0 ATP hydrolase), lifetime of the drop in IMM potential during OGD (ψm decay) and the recovery of IMM potential after OGD-reoxygenation (ψm

recovery). This shows that, regarding the 7 experimental variables, the phenotypes of cells double knock-down MFN2/ANT2 and MFN2/IF1 are reverted along the first PC as compared to single MFN2 KD cells. However, while MFN2/ANT2 phenotype is similar to the one of control cells, MFN2/IF1 phenotype diverge from control cells along the second PC. In conclusion, both ANT2 and IF1 KD did not reverted as efficiently as the MFN2 KD phenotype, showing a major effect for ANT2 and a partial effect for IF1. (B) Percentage of cell death type after 4 h OGD and 2 h reoxygenation in siControl measured by FACS with a caspase staining (early apoptosis) and double staining with propidium iodide and caspase staining (late apoptosis/necrosis).

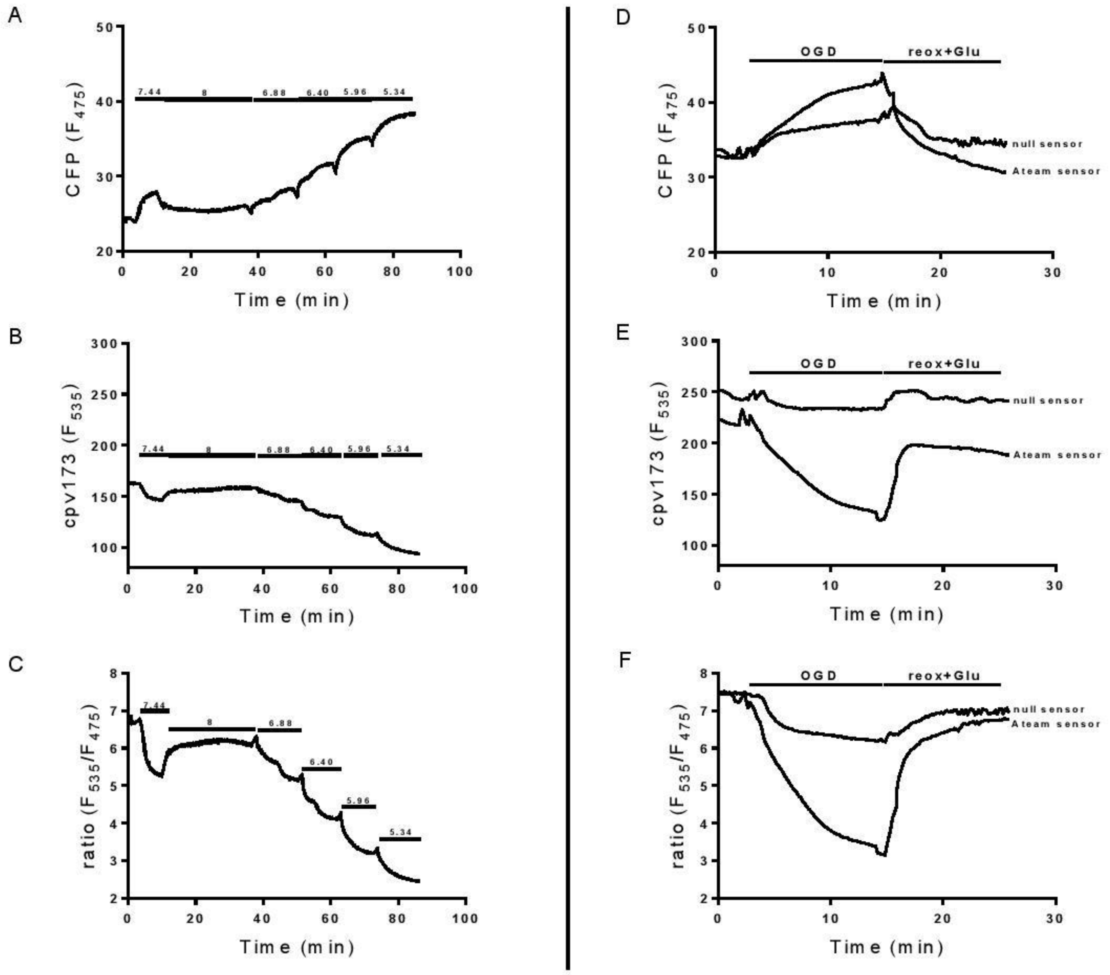

Figure S6*: pH sensitivity of cameleon fluorescent protein.*

(A) Panels A, B, C show the calibration of a cameleon fluorescent proteins without the ligand domain (null sensor) by increasing pH of solutions buffered from 5.5 to 8 in order to estimate the YFP-based biosensors in OGD experiments. Panels D, E and F show a comparison of the null sensor and the native cytosolic Ateam cameleon fluorescent protein (Ateam sensor) in cells subjected to 15min oxygen glucose deprivation (OGD) and to reoxygenation (reox+Glucose). CFP fluorescence (CFP (F_{475})), FRET channel (CFP (F_{535})) and ratio FRET/CFP are shown in panels A/D, B/E and C/F, respectively. Acquisitions have been performed with the same lamp intensity and exposition applied for all experiments in this paper.
